# Assessing financial risks of foreign agricultural investment in belt and road countries: A risk index approach and VHSD-EM model analysis

**DOI:** 10.1371/journal.pone.0293146

**Published:** 2023-12-20

**Authors:** Ying Tian, Fayaz Hussain Tunio

**Affiliations:** 1 Institute of Agricultural Economics and Development, Chinese Academ y of Agricultural Sciences, Beijing, China; 2 Department of Law, Shaheed Zulfiqar Ali Bhutto University of Law, Karachi, Pakistan; Hosei University: Hosei Daigaku, JAPAN

## Abstract

This study establishes a risk index system to evaluate the financial risks of foreign agricultural investment in Belt and Road countries. Agricultural foreign investment risk prevention has emerged as a crucial concern across various sectors globally. We assess the four key dimensions such as political and military risk, economic market risk, social and cultural risk, and resource and environmental risk. We employ the Vertical and Horizontal Leveling Method and Entropy Weighting Method (VHSD-EM) for measuring and analyzing foreign agricultural investment risk levels in Belt and Road countries from 2014 to 2021. Moreover, we used spatial correlation analysis, the Getis-Ord Gi* statistic, to identify hot and cold spots of agricultural foreign investment risks. First political & military, and environmental risks are the main influencing factors of agricultural foreign investment risk. The average AFDI level exceeded in Southeast and South Asia, and certain spillover effects were found in Southeast Asia. Second, the Belt and Road" initiative effectively reduces the risk of AFDI and helps to weaken the spillover effect among fellow countries. A significant spillover effect in AFDI from neighboring countries can lead to high-risk areas for sustained AFDI formation. Third to address such challenges, the Chinese government has prerequisites to enhance foreign agricultural investments in Belt and Road countries and establish a measurement index for agricultural investment risks. Government needs to establish a public service system to enhance the development of large-scale multinational agricultural enterprises. Foreign cooperation is essential for multi and bilateral investment negotiation and optimizing the financial tools to mitigate agricultural foreign direct investment risk in Belt and Road countries.

## 1. Introduction and literature review

The Belt and Road cooperation initiative has launched a series of work with economic construction as the core, which brings not only great opportunities for Chinese agricultural enterprises to invest abroad but encourages Chinese enterprises to invest more in foreign agriculture in Belt and Road countries and also promotes Chinese enterprises to increase the amount of foreign direct agricultural investment. After China launched the Belt and Road initiative in 2013, China’s foreign direct investment rapidly grew in member countries. Among them, foreign investment in agriculture increased from US $3.956 billion in 2013 to US $27.115 billion in 2021. As a proactive global agricultural resource allocation behavior, agricultural investment can help developing countries solve funding shortages for agricultural development, ensure a stable domestic food supply, and promote the agricultural development of host countries. China’s agriculture has important practical significance globally in the Belt and Road initiative context. It can enhance the international pricing power of agricultural products, improve agricultural production efficiency, alleviate the pressure on domestic agricultural resources, and promote the structural reform of the agricultural supply side.

The uncertainty of the international economic and trade environment is increasing sharply. The United States and other Western countries continue to implement the policy of "De-Sinicization," tighten the review system of foreign investment, and issue the Act on Promoting Agricultural Security and Safety. The risks of foreign investment in China’s transnational agricultural enterprises will continue to expand. The launch of the "Blue Dot Network" program of the United States has disrupted China’s strategic layout of the Belt and Road, further increasing the uncertainty of agricultural investment risks in Belt and Road countries. However, China’s agriculture is expand globally but facing practical challenges, such as insufficient agricultural technology, the small scale of overseas agricultural investment, and the high risk of overseas agricultural investment. A complex and changeable investment environment creates the difference in interest demands of Belt and Road countries. It raises the risk of failure in agricultural investment for Chinese firms. The spatial correlation of China’s direct investment in Belt and Road countries objectively exists, and spatial distribution is not completely random but has significant spatial dependence on high spatial agglomeration characteristics [1]. Any economy’s outward agricultural investment depends on its investment needs and some essential conditions likewise political & military, economic market, social & cultural, and resource & environment of the host country. By the end of 2021, China’s agricultural investment projects, i.e., Belt and Road, have reached 13.68 billion US dollars, recorded for 7.50% of the total. Excessive investment risks have directly affected China’s investment in Belt and Road countries. How is the Belt and Road initiative effective and sustainable in reducing agricultural investment risks? Do any investment risks relate to Belt and Road countries in construction spatially? The examination of these issues serves to enhance our comprehension of the evolving dynamics surrounding agricultural investment as a pivotal convergence point for countries along the Belt and Road initiative, fostering a shared community of interests and a collective vision for the future. This analysis also holds profound practical implications and a catalytic role in advancing China’s proactive pursuit of agricultural globalization, expediting the maturation of contemporary agricultural practices, enhancing agricultural productivity and effectiveness, and bolstering international competitiveness. Investment risks along the Belt and Road are mainly divided into two categories; (i) descriptive research, and existing studies have used risk assessment methods such as the "difference-in-differences" analysis [2], the method of moments analysis [3], and the comprehensive evaluation method [4], spatial autocorrelation, principal component analysis [5], grey correlation method [6], and entropy method [7] to conduct in-depth analysis from political, economic, and social perspectives. (ii) This empirical investigation employs econometric models in quantitative research, consistently indicating that emerging economies hold greater allure for China’s Outward Foreign Direct Investment (OFDI).

Within the realm of foreign direct investment (FDI), the influence of political risks has garnered the attention of scholars. [2] have postulated that political risk can serve as an attractive magnet for foreign direct investment inflows. In contrast, an opposing school of thought argues that reduced political risks do not necessarily foster favorable conditions for foreign direct investment. [3] have provided empirical evidence suggesting that political risks tend to displace foreign direct investment. Furthermore, they have highlighted the growing significance of the Bilateral Investment Treaty system, particularly in relation to the proportion of foreign direct investment outflow within a national economy.

Extending the analysis to spatial dimensions, [1] incorporated a spatial weight matrix in their research to examine the impact of geopolitical risks on the geographical distribution of China’s direct investment in Belt and Road countries. Their findings underscored a significant spatial correlation between China’s investment patterns and the Belt and Road initiative. Notably, violent geopolitical risks were found to exert a pronounced adverse effect on China’s foreign direct investment. [8] adopting diverse measurement methodologies, explored overseas investment risk factors. Their investigation revealed that a company’s overseas investment is influenced by social conditions and the business environment, whereas a country’s investment risk is shaped by the intricate interplay of political and economic factors.

Furthermore, [9] assessed the investment risk levels of 50 Belt and Road countries from 2014 to 2017. They accomplished this by constructing an investment risk system specific to Belt and Road countries and employing the VHSD-EM model. While their study provided insights into the evolving trends of investment risk within these countries and regions over a four-year span, it did not delve into the spatial correlation analysis of investment risks along the Belt and Road initiative. Additionally, the study did not further investigate the distribution of cold and hot spots regarding agricultural foreign investment risks. Research in the domain of agricultural outward investment has undertaken comprehensive investigations into various related facets, such as agricultural investment risks [10], food security [11], issues pertinent to agricultural outward investment [12], factors influencing agricultural investment [13], and global agricultural resource utilization [14]. However, the focus on China’s agricultural outward investment remains relatively limited. A majority of these studies center on the current landscape of China’s agricultural globalization efforts [12], the agricultural investment environment in countries along the "Belt and Road" initiative [15], risk assessment for agricultural investments along the "Belt and Road" [16], location choices for China’s agricultural investment cooperation with countries along the "Belt and Road" [17], and strategies for mitigating agricultural investment risks [18].

[17,19] discovered that China’s Outward Foreign Direct Investment (OFDI) primarily gravitates toward high-risk countries within the "Belt and Road" initiative. Risk preference is discernible across political, military, and socio-cultural dimensions, while risk aversion manifests itself within economic and financial domains. [16] developed indicators to quantitatively assess the evolving trends and risk levels of agricultural investment and financial risk within countries along the Belt and Road, considering four dimensions: political, economic, social, and industrial. However, they did not directly measure investment risk. It’s important to note that this methodology relies on matrices to determine weights, limiting its ability to fully capture the information encapsulated within each indicator. [6] examined agricultural investment risks associated with China’s engagement with ASEAN countries using fuzzy comprehensive evaluation and grey correlation methods. Their findings indicated that major ASEAN countries exhibit higher agricultural investment risks, whereas countries such as Laos, Malaysia, and Indonesia demonstrate relatively lower levels of risk.

In general, previous studies on the influencing factors of investment in Belt and Road countries are relatively rich but lack measurement and spatial analysis on the risk of foreign agricultural investment. Therefore, by building a comprehensive risk assessment index system, we analyze the temporal and spatial evolution characteristics of four types of risks in Belt and Road countries from 2014 to 2021 using spatial autocorrelation, cold and hot spot analysis and provide some reference for China to prevent agricultural investment risks in Belt and Road countries.

First contribution of this research lies in its comprehensive exploration of effective methodologies for evaluating the agricultural investment risk prevalent among countries participating in the Belt and Road initiative. Utilizing the "VHSD-EM" model, this study assesses agricultural investment risk, allowing for a nuanced understanding of its dynamic evolution patterns over time. Furthermore, the article advances strategic recommendations and suggestions aimed at enhancing the measurement techniques for assessing foreign agricultural investment risk. This endeavor seeks to establish a dynamic rating system and early warning mechanism while fortifying enterprises’ capabilities to manage and withstand risks effectively.

Second, extending its purview beyond risk measurement in outward agricultural investments, this paper employs spatial correlation and cold and hot spot analyses to evaluate the spatial characteristics of investment risks within 51 Belt and Road countries. Through this analysis, it identifies nations marked by a sustained concentration of agricultural outward investment risks, thereby providing valuable decision-making insights for the accurate identification of high-risk regions and the strategic dispersion of enterprise investments.

## 2. Risk measurement and assessment of agricultural outward investment

### 2.1 Index system of agricultural foreign investment risk of belt and road countries

Considering the availability and completeness of data, the 51 Belt and Road countries: Brunei, India, Indonesia, Laos, Malaysia, the Philippines, Singapore, Thailand, Albania, Bulgaria, the Czech Republic, Hungary, Latvia, Macedonia, Montenegro, Poland, Romania, Serbia, Slovakia, Slovenia, Ukraine, Kazakhstan, Kyrgyzstan Tajikistan, Uzbekistan, Algeria, Armenia, Azerbaijan, Egypt, Georgia, Iran, Iraq, Israel, Jordan, Kuwait, Lebanon, Morocco, Oman, Qatar, Saudi Arabia, Tunisia, United Arab Emirates, Yemen, Bangladesh, Maldives, Nepal, Pakistan, Sri Lanka, Vietnam, Mongolia, Russia. The time span is from 2014 to 2021.

After removing the sample of countries with missing economic and financial risk data in some years, the missing data are interpolated by linear interpolation. To cover the indicator information as comprehensively as possible, this paper, based on the principles of comprehensiveness, practicality, representativeness, and operability, constructs a risk measurement system for countries along the " Belt and Road" covering four perspective dimensions, political & military risks, economic market risks, social & cultural risks, and resource & environmental risks, with a total of 21 basic indicators, as shown in Table 1.

**Table 1:** Agricultural investment risk index system of belt and road countries.

Risk category	Risk indicators	Indicator Description	Data sources	Attribute	Weight
**Political & Military Risks**	Political Stability	The political situation remains stable, and lake of violence or terrorism	WBGI	Positive	0.040
	Government Effectiveness	The efficiency of government policy formulation and implementation, as well as the credibility of commitments	WBGI	Positive	0.019
	Regulatory Quality	The ability of the government to establish sound regulations to promote the development of private enterprises	WBGI	Positive	0.086
	Military Intervention in Politics	The level of military involvement in host country government affairs	ICRG Political Risk Index	Reverse	0.035
	Corruption Control	The degree of government control over corruption and abuse of public power within the political system	WBGI	Positive	0.028
**Economic Market Risk**	Inflation Rate	Reflect changes in a country’s price level	WBDI	Reverse	0.082
	Per Capita GDP	Per capita production capacity	WBDI	Positive	0.084
	Economic Volatility	The fluctuation of economic growth rate in the past five years reflects the long-term stability of the economy	WBDI	Reverse	0.061
	Unemployment Rate	Unemployment situation in the host country	WBDI	Reverse	0.105
	Dependence on Foreign Trade	Reflect the degree of dependence of a country’s national economy on foreign trade	WBDI	Reverse	0.023
**Social & Cultural Risks**	National education level	Average length of education	UNESCO	positive	0.106
	Public Security Order	Number of murders per 100000 people per year	United Nations Office on Drugs and Crime	reverse	0.024
	Labor Market Regulation Situation	Minimum wage standards and working hours regulations for hiring and dismissing labor force	World Economic freedom Index	positive	0.067
	Business Regulation Situation	The difficulty of establishing a business, the restrictions on obtaining a business license, etc	World Economic freedom Index	positive	0.060
	Geographical Distance	Longitude and latitude distance between capitals	CEPII Gravity Dataset	Reverse	0.040
	Gini Coefficient	Social wealth gap	World Bank, CIA	Reverse	0.032
**Resource & Environmental Risks**	Infrastructure Quality	The quality level of infrastructure in the host country	Global Competitiveness Report	Positive	0.053
	Average Water Resource Ownership	Total freshwater resources/cultivated land area	The United Nations and Sustainable Development and Water Resources	Positive	0.011
	Agricultural Mechanization Level	Number of tractors per 100 square kilometers of cultivated land	WDI	Positive	0.024
	Per Capita Cultivated Land Area	Agricultural land area/total population	WDI	Positive	0.020

Note: The larger the value of the "positive" indicator, the smaller the risk; the smaller the value of the "reverse" indicator, the smaller the risk.

The outcomes derived from the Pearson correlation test exhibit a remarkable alignment with the findings obtained through the vertical and horizontal scaling method, as well as the entropy weighting method. Consequently, it can be inferred that the VHSD-EM model, which has been meticulously established, this research, demonstrates a noteworthy degree of accuracy. It is through this methodology that the ultimate weights assigned to each individual indicator have been determined.

### 2.2 Construction and measurement of the risk index of foreign agricultural investment of Belt and Road, countries

#### 2.2.1. Vertical and Horizontal Leveling Method (VHSD)

According to the time series function, the data of p countries, q basic indicators, and L years involved in the risk of outward agricultural investment can be arranged into a matrix, as shown below:

$$D=d_{ij}\left(t_{l}\right),i=1,2,\cdots ,p;j=1,2,\cdots ,q;l=1,2,\cdots ,L.$$ (1)

Where *D* = *d*_*ij*_(*t*_l_) represents the *j*th indicator value of the *i*-th country in year *l*. The time-series three-dimensional data of agricultural foreign investment risks of Belt and Road countries are shown in Table 2. Among them, *c*_*i*_ represents the *i*-th country.

**Table 2:** Time series three-dimensional data of agricultural foreign investment risk of Belt and Road countries.

country	*t*_1_	*t*_2_	⋯	*t*_*L*_
	*d*_1_ *d*_2_ ⋯ *d*_*q*_	*d*_1_ *d*_2_ ⋯ *d*_*q*_	**⋯**	*d*_1_ *d*_2_ ⋯ *d*_*q*_
***c***_**1**_	*d*_11_(*t*_1_)*d*_12_(*t*_1_) ⋯ *d*_1*q*_(*t*_1_)	*d*_11_(*t*_2_)*d*_12_(*t*_2_) ⋯ *d*_1*q*_(*t*_2_)	**⋯**	*d*_11_(*t*_*L*_)*d*_12_(*t*_2_) ⋯ *d*_1*q*_(*t*_*L*_)
***c***_**2**_	*d*_21_(*t*_1_)*d*_22_(*t*_1_) ⋯ *d*_2*q*_(*t*_1_)	*d*_21_(*t*_2_)*d*_22_(*t*_2_) ⋯ *d*_2*q*_(*t*_2_)	**⋯**	*d*_21_(*t*_*L*_)*d*_22_(*t*_*L*_) ⋯ *d*_2*q*_(*t*_*L*_)
**⋮**	**⋯**	**⋯**	**⋯**	**⋯**
***c***_***p***_	*d*_*p*1_(*t*_1_)*d*_*p*2_(*t*_1_) ⋯ *d*_*pq*_(*t*_1_)	*d*_*p*1_(*t*_2_)*d*_*p*2_(*t*_2_) ⋯ *d*_*pq*_(*t*_2_)	**⋯**	*d*_*p*1_(*t*_*L*_)*d*_*p*2_(*t*_*L*_) ⋯ *d*_*pq*_(*t*_*L*_)


$$y_{ijl}=\frac{d_{ijl}-d_{\bar{ijl}}}{\sigma_{ijl}},i=1,2,\cdots ,p;j=1,2,\cdots ,q;l=1,2,\cdots ,L.$$ (2)

Firstly, the value of the *j*th indicator in the *i*-th country in year *l* is standardized to obtain *y*_*ijl*_, taking the mean and standard deviation of the *j*th indicator from all countries in year *l*, obtains $d_{\bar{ijl}}$ and $d_{\bar{ijl}}$.

Secondly, the risk function for agricultural outward investment is set as:

$$P_{i}\left(t_{l}\right)=\sum_{j=1}^{p}\mu_{j}j_{ij}\left(t_{l}\right)$$ (3)

In the equation, *μ*_*j*_ is the indicator weight, *P*_*i*_(*t*_l_) is the comprehensive risk value of country *i* in year *l*. Determine the indicator weight using formula (4).


$$\sigma^{2}=\sum_{L=1}^{L}\sum_{i=1}^{p}{\left(p_{i}\left(t_{l}\right)-\bar{p}\right)}^{2}=\mu \sum_{L=1}^{L}A_{L}\mu =\mu^{T}A\mu$$ (4)

Among them, *μ* = (*μ*_1_, *μ*_2_, ⋯, *μ*_*p*_)^*T*^, $A=\sum_{L=1}^{L}A_{l}$ is a *p* order Symmetric matrix, $A=B_{l}^{T}B_{l}$, *l* = 1, 2, ⋯,*L*.

When *μ*^*T*^ *μ* = 1 and taking the eigenvector corresponding to the maximum value of A, *σ*^2^ reaches a maximum value, the weight vector *μ*_*j*_ is obtained by normalizing the feature vectors.

#### 2.2.2. Entropy Weighting Method (EM)

Using range standardization to process indicator data, if *d*_*ijl*_ is a positive value, then

$y_{ijl}=\frac{d_{ijl}-min\left(d_{ijl}\right)}{max\left(d_{ijl}\right)-min\left(d_{ijl}\right)}$; If *h*_*ijl*_ is a negative value, then

$$y_{ijl}=\frac{max\left(d_{ijl}\right)-d_{ijl}}{max\left(d_{ijl}\right)-min\left(d_{ijl}\right)}.(i=1,2,\cdots ,p;j=1,2,\cdots ,q;l=1,2,\cdots ,L)$$ (5)

Among them, the *j*th indicator of the *i*-th country obtained *y*_*ijl*_ After range normalization of the indicator value in the *l*-th year.

Calculate the degree of variation of indicator values:

$$V_{ijl}=\frac{y_{ijl}}{\sum_{i=1}^{p}y_{ijl}}$$ (6)

Among them, the characteristic proportion of the *i*-th country in the *l*-th year under the *j*th indicator is expressed as *v*_*ijl*_.

From this, calculate the EM value of the *j*th indicator, $E_{jl}=-\frac{1}{ln\left(p\right)}\sum_{i=1}^{p}v_{ijl}ln\left(v_{ijl}\right)$, Among them, when *v*_*ijl*_ = 0 or 1, set *v*_*ijl*_ *ln*(*v*_*ijl*_) = 0.

Make the coefficient of difference of the indicator *F*_*jl*_, then *F*_*jl*_ = 1 − *E*_*jl*_.

The formula for calculating the weight of indicator EM is:

$$\varphi_{jl}=\frac{F_{jl}}{\sum_{j=1}^{q}F_{jl}}$$ (7)

#### 2.2.3. Basic principles and verification of the VHSD-EM model

VHSD-EM analysis combines the indicator weights calculated by the VHSD model and the EM model to obtain the final weights and calculate the comprehensive risk value of each indicator. Use MATLAB 16.0 software to perform Spearman correlation tests on the results of VHSD and EM methods.

Weights to be determined by VHSD and EM methods *μ*_*j*_ and *φ*_*jl*_ Consists of the following matrix:

$$C_{jl}={\left(\begin{array}{cc} \mu_{1} & \varphi_{1l}\\ \vdots & \vdots \\ \mu_{q} & \varphi_{ql} \end{array}\right)}_{q\times 2}$$ (8)

The final weight of each indicator *W*_*jl*_ is created by the arithmetic mean of each row of elements in *C*_*jl*_ Namely

$$W_{jl}=\left(\mu_{j}+\varphi_{jl}\right)/2$$ (9)

The specific score for each indicator is recorded as

$$P_{i}=y_{ijl}\times W_{jl}$$ (10)

### 2.3Analysis of the overall measurement results of the investment risk level of Belt and Road countries

From the time dimension, the countries’ average agricultural investment risk level shows the fluctuation in downward-upward trend, from 7.4944 in 2014 to 7.3340 in 2021, with the decrease of 2.14%. Throughout the sampling period, notable regional disparities in agricultural investment risk levels were observed among the six regions encompassed by the "Belt and Road" initiative. Specifically, the agricultural investment risk within Central and Eastern Europe, West Asia, and North Africa exhibited a distinctive temporal pattern characterized by successive phases of escalation, de-escalation, resurgence, re-escalation, and subsequent de-escalation. In contrast, the agricultural investment risk within Central Asia, Southeast Asia, and South Asia followed a discernible trend characterized by cyclical fluctuations, involving phases of decline, resurgence, decline, resurgence, and once more, decline.

In the year 2021, an intriguing observation emerged within the Belt and Road initiative as it pertains to agricultural investment risk. Specifically, four regions—Central Asia, Central and Eastern Europe, and Northeast Asia—recorded higher agricultural investment risk levels compared to the regional averages. This phenomenon implies the presence of an agglomeration effect associated with China’s outward agricultural investment risk. It’s noteworthy that, in 2021, Central Asia exhibited notably higher average agricultural investment risk levels compared to the other regions, underscoring regional variations in risk.

Moreover, substantial disparities were evident when examining the agricultural investment risk levels among the 51 countries along the Belt and Road initiative in 2021. Pakistan emerged as the country with the highest agricultural investment risk level, while Brunei ranked as the country with the lowest risk level. Notably, the disparity in the agricultural investment risk scores between these two countries was 1.80 times, signifying a significant divergence in the risk profiles of agricultural investments across different nations.

Within each region, specific countries stood out in terms of their agricultural investment risk levels. For instance, Macedonia ranked first in Central and Eastern Europe, Tajikistan claimed the top spot in Central Asia, Morocco led the pack in West Asia and North Africa, Pakistan took the lead in South Asia, Thailand held the highest rank in Southeast Asia, and Russia garnered the top position in Northeast Asia regarding agricultural investment risk levels. These regional and country-specific variations contribute to a more nuanced understanding of the complexities surrounding agricultural investment risks within the Belt and Road initiative.

Further, this paper conducts a multi-dimensional analysis of the agricultural investment risks from the perspective of temporal changes and accelerating political & military risk. At the national level, countries with higher political risks mainly include Albania and Macedonia, two Central and Eastern European countries, Southeast Asian countries such as Indonesia, and West Asian and North African countries such as Jordan. Countries in these regions generally face risks such as political instability, inefficient government, and feeble rule of law.

The economic market risk shows an increasing and deceasing trend from the national perspective, countries with high economic market risks mainly include Southeast Asian countries such as Laos and the Philippines, Central and Eastern European countries include Poland and Romania, and South Asian countries contains Vietnam and Pakistan. Among them, the fluctuations in risk levels in 2019 were mainly due to reduced inflation risk, while the decrease in risk in 2020 was mainly due to exchange rate fluctuations, that some extent indicates the economic situation along the route is quite complex and unstable factors.

The assessment of social and cultural risks within countries along the Belt and Road initiative reveals a discernible pattern characterized by an initial decline, followed by an upswing, and subsequently another decline. When viewed through a national lens, it becomes evident that countries exhibiting heightened social and cultural risks primarily encompass West Asian and North African nations, such as Morocco, Iraq, Lebanon, and Tunisia, South Asian countries, including Pakistan and India, Southeast Asian nations such as Thailand and Indonesia, and Central and Eastern European countries like Ukraine.

These countries with elevated social and cultural risks face multifaceted challenges, stemming from both internal and external sources. For instance, India and Pakistan grapple with territorial disputes, resulting in internal and external conflicts. Ukraine has been entangled in military conflicts attributable to foreign interference. Meanwhile, Iraq contends with a proclivity towards social violence and crime. The common thread among these high-risk nation lies in their intricate and evolving social landscapes, marked by disparities in social development and frequent conflicts triggered by a multitude of factors, including race, religion, and culture. These complexities underscore the importance of considering social and cultural dimensions when assessing risk within the Belt and Road initiative. The resources and environment risk shows initial rising and then relatively flat trend from a national perspective, countries with high resource and environmental risks mainly include West Asian and North African countries such as Algeria, Iraq, and Yemen, likewise South Asian countries contains Maldives, Nepal, Bangladesh, Pakistan, and Central Asian countries such as Uzbekistan. The countries with high resource and environmental risks in these countries are mainly lagging with transportation infrastructure, scarce water resources, and a small proportion of agricultural land. Such countries have weak demand for agricultural development, disadvantaged agricultural resources, and hindered overall agricultural production development, resulting in significant resource and environmental risks.

## 3. Analysis of spatial characteristics of risks in outward agricultural investment

### 3.1 Spatial correlation test

The distribution of risks in outward agricultural investment among countries is not isolated due to the strategic interaction between neighboring countries there is a significant spatial correlation in outward agricultural investment between neighboring countries. Once this spatial correlation is solidified, it may gradually form a risk accumulation area, which deserves attention. We deeply explore the spatial distribution characteristics of agricultural outward investment risks of Belt and Road countries. This paper uses *Moran’ s I* to test the spatial correlation of relevant indicators of agricultural outward investment risks. According to Tobler’s first law of geography, regions closer to each other are more closely related. Therefore, we first build a spatial weight matrix W with the following elements:

$$w_{ij}=\frac{1}{d_{ij}^{2}}$$ (11)

Among them, *d*_*ij*_ represents the geographical distance between country i and country j. Under this setting, the spatial matrix elements corresponding to distant countries are relatively small, which can identify the correlation between neighboring regions. After the spatial weight matrix is standardized, the calculation formula of Moran’s I can be expressed as:

$$Moran^{'}s\;I=\frac{\sum_{i=1}^{n}\sum_{j=1}^{n}w_{ij}\left(x_{i}-\bar{x}\right)\left(x_{j}-\bar{x}\right)}{\sum_{i=1}^{n}{\left(x_{i}-\bar{x}\right)}^{2}}$$ (12)

Where, x refers to the risk indicators of agricultural foreign investment of Belt and Road countries. If I is a positive value, it represents a positive spatial correlation between the risks of outward agricultural investment in neighboring countries, and the larger the value, the stronger the correlation; If I is a negative value, it indicates that the risk of outward agricultural investment in neighboring countries is spatially negatively correlated. The smaller the value, the greater the difference; If I is significantly 0, it indicates no spatial correlation. The Moran’s I value calculated using STATA16.0 is shown in Table 3.

**Table 3:** Global Moran’s I in the sample period.

Year	*I*	*E*(*I*)	*sd*(*I*)	*z*	p*
**2014**	0.048	-0.020	0.110	0.618	0.268
**2015**	0.184	-0.020	0.110	1.861	0.031
**2016**	0.211	-0.020	0.110	2.104	0.018
**2017**	0.268	-0.020	0.110	2.621	0.004
**2018**	0.283	-0.020	0.110	2.751	0.003
**2019**	0.293	-0.020	0.111	2.835	0.002
**2020**	0.272	-0.020	0.110	2.659	0.004
**2021**	0.249	-0.020	0.110	2.454	0.007

For the identification of spatial correlation, this paper tests the global spatial correlation of China’s agricultural outward investment risks to 51 Belt and Road countries by calculating Moran’s I index under the spatial weight matrix.

From the changing trend of China’s Moran’s I index of outward agricultural investment in the 51 countries in Table 3, we can find that: first, although China’s Moran’s I index of investment in the " Belt and Road" shows a relatively sharp upward and downward fluctuation trend, it is generally positive, which indicates that China’s agricultural outward investment in the "the Belt and Road" has a positive spatial correlation. Secondly, from the trend of the Moran’s I index over time, with 2019 as a turning point, the Moran’s I index showed a clear upward trend from 2014 to 2019 while showing a clear downward trend after 2019. It shows that before 2019, China’s spatial dependence on the overall investment in countries has gradually increased. Since 2019, this dependence has begun to weaken, and from 2021, its spatial dependence has risen again.

According to Moran’s I, the countries along the "Belt and Road" have a strong spatial agglomeration in the sample period, and there is a positive correlation between countries. After 2018, the Sample space correlation has partially increased.

**Fig 1:**
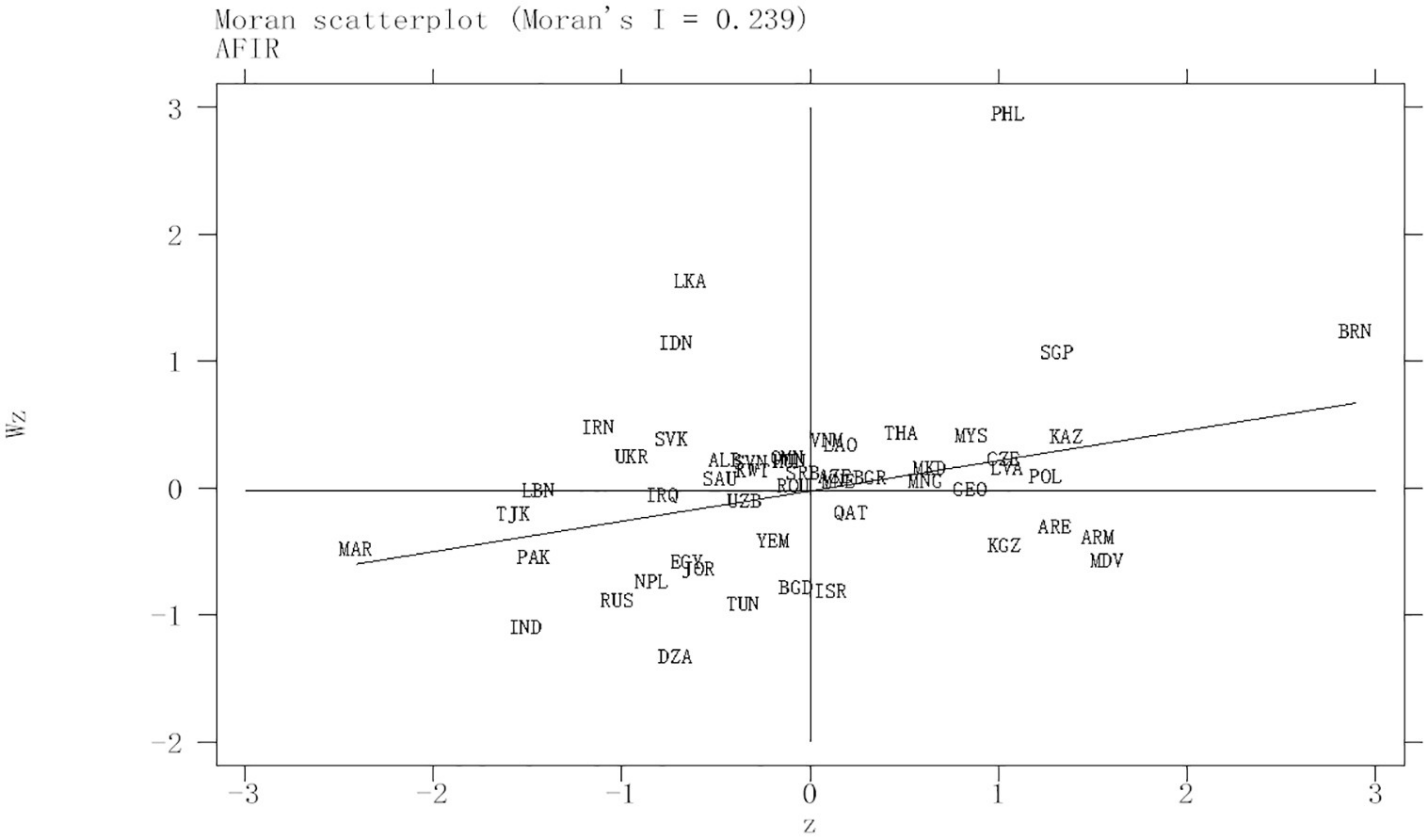
Moran scatter agricultural foreign investment risk.

In order to further observe the spatial agglomeration characteristics of China’s agricultural outward investment in countries along the "Belt and Road,” this paper draws Moran’s I scatter diagram of outward agricultural investment under the spatial weight of geographical distance, as shown in Fig 1.

It can be seen from Fig 1 that 34 countries are located in the first and third quadrants under the spatial weight of geographical distance, which further indicates that the spatial distribution of China’s agricultural outward investment in countries along the "Belt and Road" is not completely random, but highly spatial dependent. Therefore, when studying the risks of foreign investment in countries’ agriculture, we should not ignore its spatial dependence, or it will lead to biased estimates.

### 3.2 Analysis of spatial agglomeration

Due to the obvious regional characteristics of Belt and Road countries, the spatial characteristics of agricultural outward investment risks in different based on countries heterogeneous. Although Moran’s I can be used to measure spatial correlation, it is difficult to identify local characteristics of space and make an in-depth analysis from the national level. Therefore, this section further introduces the Getis-Ord statistic. $G_{i}^{*}$ Constructed by Order et al. Getis (1995), used to identify the local concentration of risks of countries along the "Belt and Road". The advantage of this unified measurement is that it can identify hot spots and cold spots in the countries, thereby describing spatial heterogeneity. The calculation formula is:

$$G_{i}^{*}=\frac{\sum_{j=1}^{N}w_{ij}(d)z_{j}}{\sum_{j=1}^{N}z_{j}}$$ (13)

Where i and j represent countries, d represents geographical distance, and *w*_ij_(*d*) is the spatial weight matrix element defined according to the distance rule, *z*_*j*_ is the observed value of country j. The geographical distance is calculated according to countries’ longitude and latitude coordinates. The average distance between countries along the "Belt and Road" is found to be 5946.42 km. considering the radiation range of countries, d is set as 836 km. Therefore, *w*_ij_(*d*) can be expressed as:

$$w_{ij}\left(d\right)=\left\{\begin{array}{ll} 1, & if\;d_{ij}\leq 836\;\text{km}\\ 0, & if\;d_{ij}>836\;\text{km} \end{array}\right.$$ (14)

In Eq (3), the numerator represents the sum of values within a radius of 836 kilometers from the center point of country i, and the denominator represents the sum of values for all countries. Therefore, this unified measurement measures whether country i and its surrounding countries (i.e. countries within a radius of d kilometers) have relatively large or small ratios compared to all countries. If the statistic is high (low) at a significance level of 5%, the region is identified as a hot (cold) point. From this, spatial clustering identification of agricultural investment risks can be carried out in 51 countries.

Table 4 depicted political and military risks within the context of the Belt and Road initiative reveals certain consistent trends. Over five consecutive years, five countries have consistently been designated as hot spots: Yemen, Iraq, Thailand, Pakistan, and Algeria. Conversely, six countries have consistently been designated as cold spots during the same period: Slovakia, Czech Republic, United Arab Emirates, Latvia, Brunei, and Sri Lanka.

**Table 4:** Identification of hot and cold countries.

Risk Indicators	2014	2015	2016	2017	2018	2019	2020	2021	Last Five Years	Last Two Years
**Number of Hot spot Countries**										
**Political & Military Risk**	10	13	12	10	11	8	6	7	5	7
**Economic Market Risk**	13	13	14	12	13	10	10	10	5	8
**Social & Cultural Risk**	11	10	15	10	12	10	7	6	3	5
**Resource & Environmental Risk**	12	13	12	12	11	9	9	10	4	7
**Number of Cold spot Countries**										
**Political & Military Risk**	13	9	10	12	11	14	16	16	6	10
**Economic Market Risk**	9	9	8	10	9	12	12	12	4	7
**Social & Cultural Risk**	11	12	7	12	10	13	16	16	3	6
**Resource & Environmental Risk**	10	9	10	10	11	14	13	12	2	6

With the advancement of the Belt and Road initiative, seven countries have emerged as hot spots in recent years. These include Yemen, Iraq, Iran, Lebanon, Albania, Thailand, and Pakistan. Simultaneously, ten countries have transitioned into cold spots, such as Slovakia, Brunei, Czech Republic, United Arab Emirates, Latvia, Sri Lanka, Romania, Israel, Slovenia, and Qatar.

Evaluating economic market risks, five countries have sustained hot spot status for five consecutive years: Laos, the Philippines, Vietnam, Romania, and Poland. In contrast, four countries have consistently been identified as cold spots: Jordan, Armenia, Montenegro, and Tunisia.

In recent years, with the promotion of the Belt and Road initiative, eight countries have become hot spots, including Laos, the Philippines, Romania, Poland, Vietnam, Pakistan, Oman, and the Czech Republic. Meanwhile, seven countries have transitioned into cold spots, namely Iraq, Iran, Lebanon, Tunisia, Montenegro, Jordan, and Armenia.

Assessing social and cultural risks, three countries—Morocco, Pakistan, and India—have maintained hot spot status for five consecutive years. Concurrently, three countries—Slovakia, Kazakhstan, and the United Arab Emirates—have remained cold spots for the same duration.

With the advancement of the Belt and Road initiative, five countries have become hot spots in recent years: Morocco, Pakistan, India, Iraq, and Lebanon. In contrast, six countries have transitioned into cold spots, including Brunei, Slovakia, Kazakhstan, the United Arab Emirates, Kyrgyzstan, and the Czech Republic.

Finally, in the realm of resource and environmental risks, four countries have consistently been identified as hot spots for five years: Yemen, Iraq, Nepal, and Bangladesh. In contrast, two countries, Slovakia and the United Arab Emirates, have consistently been designated as cold spots.

With the progression of the Belt and Road initiative, seven countries have become hot spots in recent years, namely Iraq, Yemen, Algeria, Maldives, Nepal, Bangladesh, and Uzbekistan. Simultaneously, six countries have transitioned into cold spots, including Slovakia, United Arab Emirates, Hungary, Slovenia, Czech Republic, and Oman.

The distribution of agricultural investments within countries along the Belt and Road initiative exhibits a pronounced spatial agglomeration. This manifests as a core hot spot for political and military risk in West Asia and North Africa and a core cold spot for political and military risk in Central and Eastern Europe. This spatial distribution underscores the elevated overall risk levels in Belt and Road countries, particularly in terms of political and military risks.

## 4. Conclusion and discussion

This study focuses on the measurement and spatial characteristics analysis of agricultural investment risks in countries participating in the Belt and Road initiative. Firstly, it is observed that the agricultural investment risks in member countries have generally decreased over time. Political & military risks, as well as resource & environmental risks, emerge as the primary factors influencing agricultural foreign investment risks. Secondly, significant variations exist in the levels of agricultural investment risks among the six major sectors within the Belt and Road region. These sectors exhibit investment risks that surpass the average levels observed in Northeast Asia, Central and Eastern Europe, and Central Asia. Furthermore, the average risk level of agricultural investment between 2014 and 2021 far exceeds to Southeast and South Asia, although agricultural investment risks in Southeast Asia demonstrate a certain degree of spillover effect. Thirdly, there are notable discrepancies in the risk levels of agricultural investment among Belt and Road countries. In 2021, Macedonia, Tajikistan, Morocco, Pakistan, Thailand, and Russia rank highest in terms of agricultural investment risks within their respective regions. The ability of agricultural investment to withstand risks will face severe tests due to ongoing factors such as global inflation, power struggles among major countries, and the COVID-19 pandemic’s impact on the global economy. The aforementioned analysis demonstrates that the "Belt and Road" initiative effectively reduces the risks associated with agricultural investment and helps to mitigate spillover effects among neighboring countries. Finally, due to strategic interactivity in the behavior of neighboring countries, a significant spatial correlation exists in outward agricultural investment, displaying distinct spatial agglomeration characteristics. Agricultural investment from neighboring countries exhibits a substantial spillover effect, which can contribute to the formation of high-risk areas for sustained agricultural investment.

Based on the aforementioned research findings, this paper presents countermeasures and recommendations encompassing four key aspects. Firstly, it is proposed to enhance agricultural investment in countries participating in the Belt and Road initiative. China should bolster foreign agricultural cooperation with these countries, actively establish a dynamic rating and early warning mechanism for Belt and Road countries, and effectively strengthen China’s agricultural foreign-funded enterprises’ investments in these countries. To enhance investment efficiency and mitigate systematic risks, China should concentrate to optimizing the agricultural investment environment, and reinforcing the "five links" in agriculture, that facilitating the upgrade of the agricultural product supply chain, and fostering high-quality development of bilateral and multilateral cooperation. Moreover, for countries that facing distinct type risks, China needs to conduct an in-depth consultation and exchange risk affected countries. This necessitates the formulation of diverse strategic collaboration models and the enhancement of infrastructure development in conjunction with these countries. Moreover, the establishment of a cooperative mechanism to foster coordination among countries and the facilitation of the exploitation of synergistic strengths between Chinese enterprises and those in Belt and Road countries are of paramount importance.

This research proposes the creation of an index designed to evaluate agricultural investment risks within Belt and Road countries, coupled with the periodic release of rating reports on China’s Agricultural Overseas Investment Risk Index. The comprehensive evaluation of agricultural investment risks in Belt and Road countries should encompass a multitude of risk categories, encompassing political and military risks, economic and market risks, social and cultural risks, as well as resource and environmental risks. This evaluation should not only account for the aggregate level of these risks but also dissect their respective compositions and structures. Establishing a risk management model and constructing a measurement index for agricultural investment risks in countries are crucial, along with the regular release of risk measurement index reports. Simultaneously, an internal evaluation mechanism should be established to assess national risk management in host countries, emphasizing the effective combination of domestic and external evaluations within these host countries. The evaluation mechanism for national risk management should be enhanced to promote a comprehensive service platform that offers 24/7 early warning and evaluation of overseas project risks. This platform should dynamically select a blacklist of multinational agricultural investment risk enterprises and provide timely warnings and regular assessments. Collaborative mechanisms should be fostered to safeguard the interests and ensure the safety of overseas agricultural enterprises.

In order to effectively mitigate the elevated agricultural investment risks prevalent in Central and Eastern European countries, it is imperative to harness the catalytic potential of the "17+1" cooperation mechanism that exists between China and these regions. Recognizing the disparities in agricultural investment landscapes among individual nations, it becomes essential to delve into specific key industries and products tailored to each country’s unique context. Expanding the purview of agricultural cooperation is vital in order to establish a multifaceted framework for collaborative endeavors. The government should proactively facilitate national-level cooperation and explore projects in science, technology, education, and other fields under the "17+1" mechanism. Leveraging scientific and technological advancements in agricultural investment and trade, such as seed research and development, agricultural machinery innovation, and pest control, can play a administrative role in promoting agricultural investment. Furthermore, it is important to fully utilize the coordinating and promotional capabilities of agricultural cooperation mechanisms to deepen investment cooperation between China and Central and Eastern Europe. From the corporate perspective, Chinese enterprises should be encouraged to pursue global expansion and engage in investment cooperation with prominent local agricultural companies, fostering mutually beneficial outcomes. Additionally, China Import and Export Credit Insurance Corporation should consider implementing various crop insurance projects overseas to effectively address potential agricultural investment risks, including measures related to war, riots, exchange restrictions, and other challenges.

One limitation of this study is the absence of a comprehensive consideration of both macro and micro-level risks, including factors like China’s diplomatic relations and the intricacies of enterprise operations, within the framework of the risk assessment system. Furthermore, the wide-ranging and intricate nature of investment risks, coupled with the challenges in acquiring accurate and comprehensive data, has constrained our ability to provide more precise statistics at this time. As the "Belt and Road" initiative continues to gain momentum, there is an expectation that the evaluation system for risk indicators in outbound agricultural investment will undergo progressive refinement. In the future, this evolution is anticipated to facilitate more precise predictions of agricultural investment risks in countries along the "Belt and Road." Such advancements hold promise for providing decision-makers with robust support as they steer China’s high-quality development within the context of the "Belt and Road" initiative and the broader strategy of globalizing China’s agricultural sector.

## Supporting information

S1 File (RAR)
